# Can You Teach Yourself Point-of-care Ultrasound to a Level of Clinical Competency? Evaluation of a Self-directed Simulation-based Training Program

**DOI:** 10.7759/cureus.3320

**Published:** 2018-09-17

**Authors:** Fraser D Mackay, Felix Zhou, David Lewis, Jacqueline Fraser, Paul R Atkinson

**Affiliations:** 1 Family Medicine, Dalhousie University, Saint John, CAN; 2 Medical Education, Memorial University of Newfoundland, Saint John, CAN; 3 Emergency Medicine, Dalhousie University, Saint John, CAN; 4 Emergency Medicine, Saint John Regional Hospital, Saint John, CAN

**Keywords:** self-directed learning, simulation, point-of-care, ultrasound, pocus, point-of-care ultrasound, point of care ultrasound, bedside ultrasound, bedside ultrasonography, sim

## Abstract

Introduction

Self-directed learning in medical professions is established as an effective method of training in certain modalities. Furthermore, simulation technology is becoming widely used and accepted as a valid method of training for various medical skills, with ultrasound being one of the best studied. The use of point-of-care ultrasound (PoCUS) in the practice of emergency medicine is well established, and PoCUS is a core competency of the Royal College of Physicians and Surgeons of Canada emergency medicine standards. The primary goal of our study was to assess the effectiveness of a self-directed simulation-based training program for medical students, in terms of achieving competency in basic PoCUS scans.

Methods

Fourteen second-year medical students with no prior ultrasound experience were provided access to online study modules created by SonoSim ultrasound training solutions (SonoSim, Santa Monica, CA, US), covering ultrasound theory and methodology, and attended a two-hour introductory session where they were introduced to the study protocol, simulation equipment, and software. Participants then undertook self-directed ultrasound simulation training throughout the year, using the CAE Vimedix PoCUS simulator (CAE Healthcare, Sarasota, FL, US) and the SonoSim ultrasound training solution system. Upon reaching 10 (and 25) scans in each of the four categories (cardiac, abdomen, aorta, and pelvic), a triggered assessment was arranged in which participants scanned a live volunteer under the direct supervision of PoCUS-certified physicians. The physicians scored the participant attempts in terms of image acquisition, interpretation, and clinical understanding. No feedback was provided to the participants. Following the study, participants submitted feedback regarding the design of the study and were asked to rank their preferred training program protocols out of a provided list of five different options.

Results

At the first triggered assessment (after completing only 10 scans in each category), four out of 14 participants were scored as competent in the aorta scan, two out of 14 participants were competent in the pelvic scan, and none of the participants were competent in both the cardiac and abdominal scans. Only nine out of 14 participants completed the second triggered assessment (after completing 25 scans in each category). At the second assessment, only three participants were scored as competent in the aorta scan, two participants were competent in the cardiac scan, and one participant was competent in the pelvic scan. None of the 14 learners completed the final phase of the training and assessment protocol. Feedback following the termination of the study showed that none of the participants supported continuing the study protocol as designed originally, and the preferred study design consisted of a full-day introductory course with live models and simulation, followed by self-directed learning with simulation and live models until 50 scans in each category were achieved.

Conclusion

We were unable to demonstrate the achievement of competence in PoCUS in medical learners engaged in our combined self-directed simulation-based training program. This is in contrast to the considerable literature supporting self-directed learning and simulation-based learning for other skills. Feedback from faculty, curriculum integration, and alignment with clinical experience may be beneficial.

## Introduction

The use of point-of-care ultrasound (PoCUS) in the practice of emergency medicine is well established, nationally and internationally [1-4]. The International Federation for Emergency Medicine and the American College of Emergency Physicians (ACEP) state that PoCUS is an essential skill and that ultrasound training should begin early in emergency medicine residency [1-2]. PoCUS became a core competency of the Royal College of Physicians and Surgeons of Canada emergency medicine standards in 2008 [3]. While the Canadian College of Family Physicians Emergency Medicine (CCFP(EM)) residency does not include PoCUS as a core competency, the majority of CCFP(EM) residency programs do incorporate some degree of ultrasound training [4]. Thus, there is a need for ultrasound competence to be achieved at some point during emergency medical training.

Self-directed learning in medical professions is established as an effective method of training in certain modalities [5]. Furthermore, simulation technology is becoming widely used and accepted as a valid method of training for various medical skills, with ultrasound being one of the best studied [6-10]. In order to utilize PoCUS successfully in clinical practice, three distinct competencies are required: image acquisition, image interpretation, and clinical integration. It has been shown that undergraduate medical students, despite a lack of clinical experience or ultrasound education, are able to learn the basic ultrasound skills of image acquisition and interpretation through self-directed learning [8]. In addition, we have previously shown that clinicians value simulated PoCUS as part of their training [11] and that simulated PoCUS skills can be acquired relatively quickly, resulting in improved diagnostic accuracy, confidence, and precision in simulated emergency medical scenarios [12].

In Canada, certification as an independent practitioner in PoCUS can be achieved following a process described and administered by the Canadian Point of Care Ultrasound Society (CPoCUS; formerly the Canadian Emergency Ultrasound Society). This process includes an approved introductory course, after which 50 scans of each of the relevant areas (cardiac, abdomen, aorta, pelvis, and lung) must be completed. Candidates must then pass written, visual, and practical examinations. While it is possible to challenge some of the learning steps, all examinations must be performed satisfactorily.

Self-directed simulation-based learning offers many benefits, including a reduced need for standardized patients, instructors, fewer expenses associated with hiring standardized patients and instructors and providing flexibility to learners. However, the effectiveness of a self-directed simulation-based training program in PoCUS has yet to be evaluated. The primary goal of our study was to assess the effectiveness of a self-directed simulation-based training program for medical learners, in terms of competency achievement in basic PoCUS scans.

## Materials and methods

An invitation to participate in a self-directed simulation-based training program was extended to all second-year medical students at an accredited Canadian medical school. The first 14 students who responded to our invitations were enrolled to participate in the study. None of the participants had any prior ultrasound experience. All participants were provided access to online study modules created by SonoSim ultrasound training solutions (SonoSim, Santa Monica, CA, US) [13] covering ultrasound theory and methodology. Participants were required to complete five online exams, provided by ACEP [14]. There was no minimum pass mark.

Following completion of the online exams, all participants attended a two-hour introductory session where they were introduced to the study protocol, simulation equipment, and software. Participants then undertook self-directed ultrasound simulation training throughout the year, using the CAE Vimedix PoCUS simulator (CAE Healthcare, Sarasota, FL, US) [15] and the SonoSim ultrasound training solution system [13]. Participants logged each simulated scan, including duration, the simulator used, body area scanned, participant interpretation of the image, and clinical correlation. Upon reaching 10 (and 25) scans in each of the four categories (cardiac, abdomen, aorta, and pelvic), lung scans were introduced to the CPoCUS program after our study was conducted, a triggered assessment was arranged in which participants scanned a live volunteer under the direct supervision of PoCUS-certified physicians. The physicians scored the participant attempts in terms of image acquisition, interpretation, and clinical understanding. No feedback was provided to the participants.

Upon completion of training (50 scans in each category), participants were assessed using the same criteria as other physicians seeking certification as independent practitioners. The outcomes assessed were the number of participants deemed to be competent, as well as the number of scans needed to achieve competence. Following the study, participants submitted feedback regarding the design of the study and were asked to rank their preferred training program protocols out of a provided list of five different options (Appendix).

## Results

**Figure 1:**
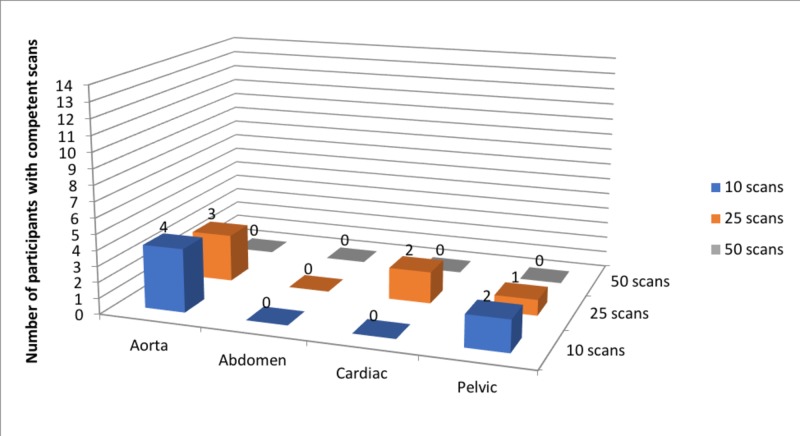
Number of participants achieving competence in each of the four basic point-of-care ultrasound scans at the three triggered assessments

All 14 learners completed the first phase of the training and assessment protocol. At the first triggered assessment (after completing only 10 scans in each category), four out of 14 participants were scored as competent in the aorta scan, two out of 14 participants were competent in the pelvic scan, and none of the participants were competent in both cardiac and abdominal scans (Figure 1). Only nine out of 14 participants completed the second triggered assessment (after completing 25 scans in each category). At the second assessment, only three participants were scored as competent in the aorta scan, two participants were competent in the cardiac scan, and one participant was competent in the pelvic scan (Figure 1). None of the 14 learners completed the final phase of the training and assessment protocol.

**Figure 2:**
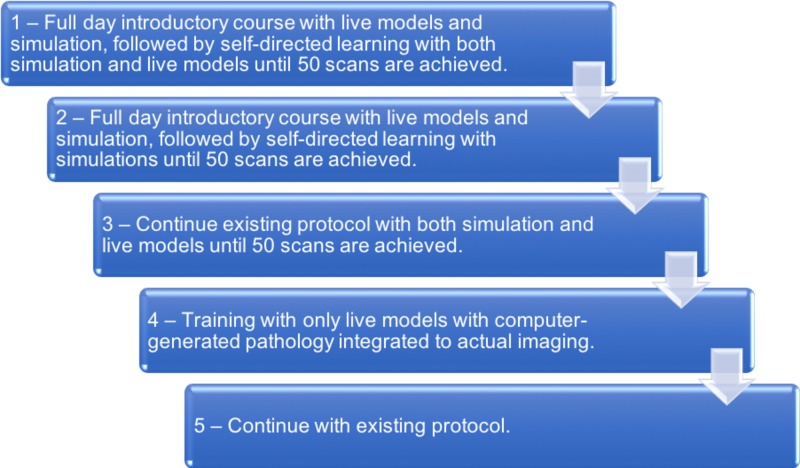
Summary of participant feedback regarding the preferred hypothetical method of completing the study, in descending order of preference

Participants cited a lack of available time and underestimating the workload as the main reasons for dropping out. Feedback following the termination of the study showed that none of the participants supported continuing the study protocol as designed originally (Figure 2). Out of the options listed, participants would have preferred a design in which a full-day introductory course with live models and simulation was followed by self-directed learning with simulation and live models until 50 scans in each category were achieved (Figure 2).

## Discussion

Participants in our study were unsuccessful in achieving competence in PoCUS from our combined self-directed simulation-based training program. The combination of self-directed and simulation-based training has obvious appeal in that it eliminates the time and expenses associated with recruiting and hiring instructors and standardized patients. In addition, allowing people to direct their own learning provides flexibility and facilitates multiple learning styles, conducive to creating competent and confident clinicians [5]. Previous research has demonstrated that undergraduate medical students can successfully learn basic PoCUS skills using simulation equipment [8]. The primary goal of our investigation was to evaluate the effectiveness of a self-directed and simulation-based training program for achieving competency in basic PoCUS scans, as tested by a standard clinical assessment process.

The study protocol was not completed, as five out of 14 students who began the study pulled out before the second triggered assessment. Participants cited a lack of available time and underestimating the workload as the main reasons for dropping out. Participants also expressed their desire for teachers who would provide feedback during their training. From the available data, we can see that although a small number of individuals were able to demonstrate competency in a limited number of modalities after only 10 simulated scans, participants did not improve in three out of four of the basic PoCUS scans assessed (Figure 1). More specifically, while two participants had improved in one of the four scans (cardiac), six participants did not demonstrate any improvement while two participants had lower scores (Figure 1). Overall, while our study protocol was not completed, our available data suggests that our combined self-directed simulation-based training program would not be sufficient to attain competence in PoCUS, despite considerable literature supporting self-directed learning and simulation, individually [5-9].

A 2006 systematic review of high-fidelity simulation in medical education [10] noted that 47% of the studies reviewed suggest feedback is the most important component of simulation-based training. In addition, 25% of the studies reviewed reported that the integration of simulation into the standard medical education curriculum was essential [10]. In our self-directed simulation-based learning program, there was no feedback at any time and participants were required to practice simulation on their time, with no integration into their medical school curriculum. Medical students tend to take on multiple commitments and time management is a skill that is often honed during, not prior to, medical school. Self-directed learning comes with its own challenges, and some participants underestimated the number of hours required to attain competence. The study protocol did not specify a time commitment beyond the stipulated number of scans required.

There are several limitations to our study. Notably, we only assessed a single training program protocol using a specific set of online modules and simulation suites. Furthermore, only nine out of 14 participants completed the second triggered assessment and no participants progressed further into the study. While it is possible that competency would have been achieved if the study protocol had been completed, both available data (Figure 1) and participant feedback (Figure 2) do not support repeating this study design. We limited the number of participants to 14 given the available resources (e.g. limited access to simulation equipment, sharing the equipment and space with a tertiary teaching hospital). This number was felt to be sufficient for a pilot study, though including a larger number of patients may have captured a wider array of learning styles and possibly have led to the study being completed. Combining two simulation systems [13,15] reduced the concern of inherent limitations in any single individual simulation suite. However, no system perfectly simulates a real patient, and participant feedback reflected this. It should be noted that the simulation equipment and suites are constantly undergoing revision and improvements, thus issues from equipment/suite limitations may be less of a concern in future studies. Based on our feedback, future research should evaluate simulation-based learning with feedback from PoCUS-certified physicians and may benefit from a larger sample size.

## Conclusions

We were unable to demonstrate the achievement of competence in PoCUS in medical learners engaged in our combined self-directed simulation-based training program. This is in contrast to the considerable literature supporting self-directed learning and simulation-based learning for other skills. Feedback from faculty, curriculum integration, and alignment with clinical experience may be beneficial.

## Appendices

Feedback questionnaire given after completion of the study

Please rank the following training program protocols in order of preference, with 1 being the most preferred and 5 being the least preferred.

- Continue with existing protocol.

- Continue existing protocol with both simulation and live models until 50 scans are achieved.

- Full-day introductory course with live models and simulation, followed by self-directed learning with both simulation and live models until 50 scans are achieved.

- Full-day introductory course with live models and simulation, followed by self-directed learning with simulations until 50 scans are achieved.

- Training with only live models with computer-generated pathology integrated to actual imaging.
